# Biodemographic Analyses of Longitudinal Data on Aging, Health, and Longevity: Recent Advances and Future Perspectives

**DOI:** 10.1155/2014/957073

**Published:** 2014-07-24

**Authors:** Konstantin G. Arbeev, Igor Akushevich, Alexander M. Kulminski, Svetlana V. Ukraintseva, Anatoliy I. Yashin

**Affiliations:** Center for Population Health and Aging, Duke University, Erwin Mill Building, 2024 W. Main Street, P.O. Box 90420, Durham, NC 27705, USA

## Abstract

Biodemography became one of the most innovative and fastest growing areas in demography. This progress is fueled by the growing variability and amount of relevant data available for analyses as well as by methodological developments allowing for addressing new research questions using new approaches that can better utilize the potential of these data. In this review paper, we summarize recent methodological advances in biodemography and their diverse practical applications. Three major topics are covered: (1) computational approaches to reconstruction of age patterns of incidence of geriatric diseases and other characteristics such as recovery rates at the population level using Medicare claims data; (2) methodological advances in genetic and genomic biodemography and applications to research on genetic determinants of longevity and health; and (3) biodemographic models for joint analyses of time-to-event data and longitudinal measurements of biomarkers collected in longitudinal studies on aging. We discuss how such data and methodology can be used in a comprehensive prediction model for joint analyses of incomplete datasets that take into account the wide spectrum of factors affecting health and mortality transitions including genetic factors and hidden mechanisms of aging-related changes in physiological variables in their dynamic connection with health and survival.

## 1. Introduction

The field of biodemography focuses on development and applications of analytic approaches aimed at integrating biological knowledge and traditional demographic methods to investigate variability in mortality and morbidity across populations and between individuals. Biodemography of aging, in particular, investigates the impact of aging on longevity and health. Although biodemography is a relatively young scientific discipline, it rapidly became one of the most innovative and fastest growing areas in demography with a history of substantial achievements up to date and with great opportunities and new promises for the future [1–8]. This progress is fueled by the growing variability and amount of relevant data available for analyses as well as by methodological developments allowing for addressing new research questions using new approaches that can better utilize the potential of these data.

In this review paper, we summarize recent publications by our research group that contributed both to methodological advances in biodemography and their diverse practical applications. Three major topics are covered. Section 2.1 discusses recent developments in computational approaches to reconstruction of age patterns of incidence of geriatric diseases (as well as other characteristics such as recovery rates) at the population level using Medicare claims data. Section 2.2 reviews our recent papers on genetic and genomic biodemography, addressing both methodological advances and applications to research on genetic determinants of longevity and health. Section 2.3 summarizes recent advances in biodemographic models for joint analyses of time-to-event data and longitudinal measurements of biomarkers collected in longitudinal studies on aging. Section 3 provides concluding remarks and discusses further perspectives of research in this area.

## 2. Biodemography of Aging: Recent Theoretical and Practical Developments

### 2.1. Evaluation of Patterns and Trends in Health-Related Characteristics of Elderly at Population Level from Administrative Data on Health Services

#### 2.1.1. Age Patterns of Incidence of Geriatric Diseases at Population Level

Human Mortality Database (http://www.mortality.org/) emerged as a major source of detailed mortality and population data for researchers and others interested in human mortality and longevity. Currently, age- and sex-specific death and population counts, death rates, and life table data are provided for 37 countries. The availability of data for the entire populations in these countries over long time periods (sometimes spanning centuries, as in case of Scandinavian countries) provides excellent opportunities for evaluating trends over time and birth cohorts in age- and sex-specific mortality rates and predicting mortality patterns for populations as a whole as well as for specific subpopulations that became a hot topic for scientific research and a focus of public attention. Trends in mortality and health in rapidly growing elderly populations in developed countries are one of such topics that attract considerable interest of policymakers, governmental institutions, and health insurance organizations. Similarly to mortality analyses, evaluation of age- and sex-specific patterns and time trends in incidence of aging-related diseases in the elderly populations requires large population-based datasets with information on ages at onset of such diseases. However, such data are costly to collect at a population level and, therefore, population-based studies on patterns and trends in incidence rates are not common. A possible solution is to use data from the national registries linked to administrative data on health care services. One example of such registries in the USA is the Surveillance, Epidemiology, and End Results (SEER) data linked to the Medicare Files of Service Use (SEER-M). SEER data *per se* is an invaluable source of information on cancer statistics in the USA currently covering about 26% of the US population. Its linkage to the Medicare data provides a unique opportunity for evaluation of incidence rates of aging-related diseases in a population-based sample of the elderly (65 years and older) US individuals covered by Medicare. Medicare enrollees represent the vast majority of the US 65+ population. According to [9], about 93–96% of the US 65+ population was covered by Medicare in years 1999–2011. Thus, these data provide a unique opportunity for evaluation of incidence rates of aging-related diseases in the elderly US population with excellent statistical accuracy that cannot be achieved in analyses of other datasets. However, the possibility of selection bias cannot be completely ruled out. One still needs to utilize available information on coverage by health maintenance organizations (HMO) in computational algorithm and/or sensitivity analyses. Below we review our recent publications which presented such an algorithm for calculation of incidence rates from Medicare data and also discuss its applications in different settings.

Despite availability of this source of information for use in scientific research, such analyses have never been performed until recently. In [10], for the first time, we evaluated age patterns of incidence of common geriatric diseases at the national level using the SEER-M data. The SEER-M dataset included Medicare records for individuals diagnosed with four specific cancers (breast, colon, lung, and prostate) and skin melanoma, as well as Medicare records for the control population represented by a random 5% sample of Medicare beneficiaries residing in the SEER areas who had none of the abovementioned cancers. Altogether, Medicare records for more than 2,000,000 individuals were available for the study. We developed a computational algorithm for reconstruction of personal histories of diseases from individual records of Medicare claims containing information on dates of medical services and procedures, related International Classification of Diseases, Ninth Revision (ICD-9) diagnoses codes, and information on coverage by Medicare Part A and/or Part B and HMO in each month of respective year. A special procedure was applied for separation of the incident and prevalent cases. This algorithm was applied to evaluate age patterns of incidence rates for nineteen diseases with high prevalence in the elderly adults. They represent all major groups of chronic diseases in elderly adults such as (i) cardio-and cerebrovascular diseases (myocardial infarction, angina pectoris, stroke, and heart failure), (ii) malignancy (cancers of lung, colon, breast, and prostate, and skin melanoma), (iii) neurodegenerative diseases (Parkinson’s and Alzheimer’s diseases), (iv) pulmonary diseases (chronic obstructive pulmonary disease, asthma, and emphysema), (v) endocrine and metabolic diseases (diabetes mellitus and goiter), and (vi) miscellaneous (chronic renal diseases with renal failure, ulcer, and arthritis).

The evaluated incidence rates revealed different age patterns that can generally be classified into several types. The first was observed for myocardial infarction, stroke, heart failure, ulcer, and Alzheimer’s disease: a monotonic increase until age 85 to 95, with a subsequent slowing down, leveling off, and decline at age 100. The second type is represented by cancers of the lung and colon, Parkinson’s disease, ulcer, and renal failure: an earlier maximum and a more-symmetric shape (i.e., an inverted U-shape). The third shape type was found for the majority of diseases (e.g., prostate cancer, asthma, and diabetes mellitus): a monotonic decline or a decline after a short period of increase (two other diseases, melanoma and emphysema, can also be thought to fit into this pattern, although their age patterns could also be considered flat). These diverse patterns of incidence rates can make important implications for projections of future burden of diseases and associated medical costs.

It is important to note that analyzing large-scale administrative datasets (such as SEER-M) is not without pitfalls that might affect calculation of incidence rates and produce systematic over- or underestimation of the number of diagnoses and/or ages at onset (e.g., incorrect dates of disease onset, latent disenrollment, and incorrect reporting of date of birth/death, etc.). Therefore, extensive sensitivity analyses are necessary to check whether there is possible methodological bias in calculation of the rates. In [10] we performed detailed sensitivity analyses with different definitions of disease onset and several alternative censoring schemes used to define individual observation periods and found that the patterns are qualitatively similar in all cases. See also [11] where we thoroughly investigated alternative approaches to definition of ages at onset of different diseases that can be applied to extract this information from large administrative databases such as Medicare. We observed that, although there is variability in the rates in different scenarios (e.g., using different Medicare sources), the estimates of age-adjusted incidence rates were stable in terms of male/female ratios, time trends, and ratios of the rates for different diseases.

Another option for calculation of population-based incidence rates is to use data from the surveys representative of the population of interest and generalize the results obtained for survey respondents to the national level using individual sampling weights to get estimates at the national level. This is the distinct feature of such analyses that distinguishes them from common analyses of incidence rates that use different local or specific datasets that are not representative at the population level. The results of such common analyses, despite providing insights into the patterns of the rates in these specific samples, cannot be generalized to the population level as they do not represent the spectrum of characteristics (such as age, race, male/female ratio, etc.) of the entire population.

The National Long-Term Care Survey (NLTCS) which focuses on the US elderly (65 years and older) population has excellent design for the purposes of calculation of population-based incidence rates because it includes individual sampling weights that can be used to make population-based inference (see discussion in [12]) and it is linked to the Medicare data providing a continuous record of individual health-related information. In [10] we compared incidence rates calculated from the SEER-M data with those calculated from the NLTCS linked to Medicare data (NLTCS-M) using the same algorithm. We observed both a qualitative (i.e., the shape of age patterns of incidence rates) and quantitative (i.e., the magnitude of the rates) agreement for almost all diseases in these two datasets. The rates also showed a good agreement with incidence rates evaluated in other studies, both in the USA and in other countries. These observations suggest that the age-specific incidence rates can be adequately estimated from the Medicare files. Thus, these data and the developed computational algorithm provide an opportunity to evaluate age patterns of incidence rates for the US elderly population at the national level with unprecedented statistical accuracy and stability with respect to systematic biases. This is especially useful for the diseases with high prevalence and health care burden for which there are no available nationally representative data on incidence.

Heart disease and stroke is a good example of such diseases. They have been the leading causes of death and major causes of disability in the USA for a long time period with tremendous accumulated health care expenditures. However, no nationally representative data on incidence of acute coronary or stroke events are available neither in the inpatient nor outpatient settings. In [12] we evaluated age-specific as well as age-adjusted incidence rates of circulatory diseases such as acute coronary heart disease (that includes myocardial infarction and angina pectoris), stroke, and heart failure, in the US elderly population using the NLTCS-M data. In [11] similar analyses were performed for other diseases that represent major groups of chronic conditions in the elderly including cancer (breast, prostate, lung, and colon cancers and melanoma), neurodegenerative disorders (Parkinson’s disease and Alzheimer’s disease), diabetes, and asthma. These population-based estimates provide valuable information for analyzing the national health trends and forecasting future Medicare expenditures related to these diseases.

#### 2.1.2. Population-Based Time Trends in Incidence of Aging-Related Diseases

The algorithm for evaluation of ages at onset developed in [10] can be applied to evaluate time trends in incidence rates. In [13] we evaluated time trends in age-adjusted incidence rates of 19 aging-related diseases in the US elderly population using the NLTCS-M and SEER-M data. The results show dramatic increase in incidence rates of melanoma, goiter, chronic renal, and Alzheimer’s disease in 1992–2005, and less prominent increase for diabetes and lung cancer. Several diseases (angina pectoris, chronic obstructive pulmonary disease, and ulcer) showed a remarkable decrease in incidence rates and less dramatic decrease was found for carcinomas of colon and prostate, stroke, hip fracture, and asthma. Incidence rates of the other diseases (female breast carcinoma, myocardial infarction, Parkinson’s disease, and rheumatoid arthritis) were almost stable. We observed an excellent agreement between incidence rates in the NLTCS-M and SEER-M data for most diseases and the stability of the evaluated time trends was confirmed in sensitivity analyses. This study confirmed time trends in aging-related diseases observed in the literature but also obtained new information on trends of asthma, ulcer, and goiter (see above) among the US elderly using a nationally representative dataset. Although several studies reported trends for asthma and ulcer in the general population or among children and young adults, they did not provide trends among the elderly. Also, studies on trends in incidence of goiter in the US elderly are not available (see more details and discussion of the findings in [13]).

These time trends can be used for projections of future incidence rates for the selected diseases under the “current trend” scenario (i.e., that the current dynamics continues into the future) and respective projections of future Medicare cost associated with these diseases. However, as discussed later in this section, several other contributing factors (such as the rates of and trends in disability, comorbidity, and recovery/long-term remission) can play a significant role in these projections, thus necessitating evaluation of these factors from the data and their inclusion into a comprehensive forecasting model.

#### 2.1.3. The Impact of Disability and Comorbidity on Incidence of Aging-Related Diseases in the Elderly

One of the important issues to consider when analyzing and projecting the patterns of incidence rates in the elderly populations is disability. Different factors can influence the development and progression of disability as discussed in the literature. In particular, there is evidence that the disablement process can be accelerated in the elderly not only because of internal aging-related changes but also because of external factors. For example, such social factors as social networks, social participation, and emotional support from family and friends were shown to be associated with the disability process in the elderly people of different cultural backgrounds [14–16]. Individuals with a single or multiple chronic diseases may develop disability of varying severity. Also, individuals having different disability levels (e.g., non-disabled, mild or severe disability) may have different risks of developing certain diseases. Therefore, incidence rates for such groups of individuals will differ. However, knowledge on how incidence of chronic diseases is affected by disability in the elderly at the national level is sparse. The NLTCS-M data provide detailed information on disability and also allow reconstructing incidence rates from the linked Medicare data thus allowing one to perform such analyses. In [11, 12] we presented disability-specific patterns of incidence rates of major diseases mentioned above showing that, indeed, the risks are affected by disability levels. For some diseases (e.g., heart failure, diabetes, asthma, and Parkinson’s disease) the incidence rates were higher among individuals with severe disabilities, while for others (such as breast and prostate cancers) the higher rate was observed among people with minor disabilities. Interestingly, for many diseases institutionalized individuals have lower rates and for several (such as melanoma, lung cancer (males), colon cancer, and asthma) the lowest rates among all other disability groups including nondisabled individuals. This indicates the importance of taking trends in disability and institutionalization into account in projecting trends in incidence rates of chronic diseases in the US elderly population.

Aging individuals tend to accumulate multiple chronic conditions over their life course and multimorbidity affects chances of death and the levels of diseases-related medical expenditures. In analyses of incidence rates for separate diseases and projections of future disease burden and associated medical expenditures it is common to assume that the risks of different diseases are independent. While this assumption may be true for many diseases, it is possible for other groups of diseases to be interrelated so that the presence of one disease can affect the risk of onset of the second disease. There might be multiple reasons for that, for example, common risk factors, genetic predisposition, effects of treatment of the earlier disease on the risk of the later disease, and so forth. Evaluation of such dependencies between the aging-related diseases is important from both population and individual patients points of view. From the public health and policy perspective, the knowledge on dependent risks of diseases can help develop more accurate predictions of population mortality and morbidity including incidence and prevalence of diseases and associated medical costs. From the individual’s perspective, understanding the possible underlying causes of dependence between disease risks can be used for developing new preventive strategies and therapeutic approaches that can be implemented in clinical practice. Although several pairs of dependent diseases are known from the literature (see, e.g., [17–19]), systematic investigation of disease dependence at the national level was lacking until recently. The Medicare-based data represent an excellent and underexplored opportunity for systematic investigation of the mutual dependence of aging-associated diseases at the national level. The computational algorithm for identification of the dates of onset of diseases for Medicare beneficiaries developed in [10] can be applied for this purpose.

In [20] we evaluated such dependencies for the US elderly population using SEER-M data considering pairs of 21 diseases representing various systems in human organism. The list of diseases and the detailed discussion of the found relationships between the diseases (many of them were not observed before) can be found in [20]. Here we focus on three important implications of these observations. First, we found that the presence of earlier diagnosed disease can substantially and significantly increase the risk of developing another disease (compared to the general population), but several other pairs of diseases manifested the “trade-off” effects in which case the individuals with diagnosed disease have lower risk of getting another disease later in life. The presence of such “direct” and “inverse” dependencies among the risks of diseases can substantially alter demographic predictions of the potential gain in life expectancy due to eradication of one disease because such predictions are usually calculated using the independence of diseases assumption. For example, in case of a trade-off between cancer and some other disease individuals susceptible to cancer are less susceptible to that second disease so that those individuals saved from cancer will have less chances of developing the second disease resulting in some additional years of life expectancy (see more discussion in [21]). Second, the presence of the inverse dependencies among diseases calls attention to finding possible biological mechanisms explaining these observations. The presence of the genetic trade-offs, that is, when the same allele confers the risk of one disease but protects against the other, has been found in recent studies (see, e.g., [22]). We will review our recent findings on the genetic trade-offs and age-dependence of effects of genes in Section 2.2. Third, dependence between risks of diseases (as well as between risks of diseases and death) has methodological implications necessitating the use of the model with dependent competing risks. We will present our recent developments of such models that incorporate individual observations from longitudinal studies in Section 2.3.

#### 2.1.4. Evaluation of Recovery/Long-Term Remission and Patients’ Survival Rates at Population Level

Trends in incidence rates of aging-related diseases are important contributors to the observed trends in life expectancy and healthy lifespan of the elderly population as well as in prevalence of diseases and associated medical costs. However, forecasting future trends in these characteristics based solely on incidence of diseases can produce erroneous conclusions because it ignores trends in other contributing components such as recovery/ long-term remission and patients’ survival from the diseased state. Indeed, for example, declining incidence rates and improvement in patients’ survival can produce a complicated picture in disease prevalence and medical costs depending on which factor contributes more (e.g., increasing burden of disease prevalence and associated costs). Also, the majority of traditional forecasting models ignore the possibility of recovery/remission and individuals are usually considered ill since the age at onset of a disease until death. This can mask the effects of successful medical treatment on patients’ health and lead to the effects of “decompression of morbidity.” Therefore, understanding the entire picture of health patterns in the elderly requires evaluating population-based rates of recovery/remission and patients’ survival in this subpopulation. The tremendous research potential of the Medicare data to shed light on these topics remained unexplored until recently.

In [23] we used the NLTCS-M and SEER-M data to investigate the properties of recovery and survival rates from eleven aging-related diseases. In this study we defined recovery state not as a complete restoration of functioning of all systems and complete social rehabilitation but rather the absence of records on the disease-related medical services coded by respective ICD-9 codes for a particular time period. We considered individuals with a diagnosed disease who are staying in such health conditions that they do not need to ask for medical services associated with the disease for a long time period (e.g., one year or more). For acute conditions (e.g., myocardial infarction) this state is associated with recovery and for chronic conditions (such as asthma) it is associated with long-term remission. We developed an algorithm to extract information on such recovery/remission state from Medicare records (see also our earlier study [24] focusing on trends in survival and recovery from stroke). We found that the risk of death was significantly lower for the cohort of recovered individuals (compared to nonrecovered ones, i.e., those who did not have long periods without the appearance of new records with ICD-9 codes of the given disease) and the results were similar for the two datasets used in the analyses and for different recovery times. However, comparison of the survival of recovered individuals to the survival of the general population allowed us to conclude that recovered individuals had higher death rates than in general population for all considered diseases; therefore, the complete recovery does not occur.

The multistate models that explicitly allow for transitions between unhealthy and recovery states have long been discussed in the literature [25, 26]. However, the estimates of the model parameters from nationally representative datasets and comprehensive analyses of the properties of recovery rates have not been performed largely due to the scarcity of such data on disease onset and recovery. Therefore, the results obtained in [23] based on the two large nationally representative datasets are valuable and timely. These findings suggest that the multistate forecasting models should have a separate “recovery” state and that the mortality rates for individuals in this state cannot be equaled to those in the unhealthy state as well as to the rates of healthy individuals. Similarly to the situation with incidence rates, comorbidity should have impact on recovery rates. Therefore, future directions involve detailed analyses of related comorbidities and the analysis of their effects on recovery rates and subsequent survival.

#### 2.1.5. Population-Based Estimates of Trajectories of Medical Costs Associated with Onset of Aging-Related Diseases in the Elderly

The detailed and comprehensive analyses of relationships between Medicare costs and disability and morbidity were recently performed using the Future Elderly Model (FEM) that was developed to predict the medical costs and health status for the elderly [27]. The elaborated microsimulation forecasting procedure allows analyses of scenarios of future development (formulated by the panels of experts) affecting rates of health transitions. However, the computations were based on smaller data (compared to the SEER-M or 5% Medicare data) from the Medicare Current Beneficiary Survey (MCBS). Also, additional research is needed to evaluate the relationships between changes in individual health histories with dynamics of Medicare expenditures as the person ages. While the patterns of the medical cost increase in the last years of life were extensively investigated in the literature (see, e.g., [28]), the question on patterns of the medical costs during the time periods comprising the date of onset of chronic diseases received less attention.

In [29] we evaluated population-based estimates of trajectories of medical costs associated with onset of twelve highly prevalent aging-related diseases in the elderly using the NLTCS-M data. We found that the time patterns of the medical costs trajectories were similar for all considered diseases and can be described in terms of four easily interpretable components: (i) the prediagnosis cost associated with initial comorbidity represented by medical expenditures; (ii) the cost associated with the onset of the disease; (iii) a reduction in medical expenditures after the disease onset; and (iv) the difference between the post- and prediagnosis cost levels associated with the acquired comorbidity due to the considered disease. The trajectories were described by the model which explicitly involves four parameters reflecting these four components. The advantage of the developed model compared to the existing models of medical cost projections which are usually based on the regression models of a number of independent predictors is that it is based on aggregating health state information into a small number of covariates (or just a single covariate) determinative in predicting the risk of a health event (such as disease incidence) and whose dynamics can be determined by the model assumptions. This leads to substantial reduction of degrees of freedom compared to the existing forecasting models and, as a result, the forecasting models in continuous time estimated with the limited information might become a close achievement. This model can serve as a building block in constructing a more precise and comprehensive forecasting model of medical costs (including Medicare spending) at a population level. It is possible to extend the model to forecast health/incidence, mortality, and associated medical costs in the US elderly using even a limited set of parameters, with a great potential for improvements when more detailed data becomes available. See detailed discussion on a general comprehensive microsimulation forecasting framework in [29].

### 2.2. Genetic Determinants of Health and Longevity from Biodemographic Perspectives

#### 2.2.1. How Genetic-Demographic Models Can Improve Power of Genetic Analyses

The contemporary rapid advances in genetics provide tremendous opportunities and challenges for the field of biodemography and there is apparent need to integrate the principles of genetics and genomics into biodemography [2, 6], or, vice versa, to apply (bio) demographic principles in genetic analyses. The ongoing incorporation of genetic information into longitudinal studies is viewed as potentially “the most revolutionary element of the addition of biological data in large-scale surveys” [30] that will “increasingly provide analyses of the interactions of genetic, biological, social, economic, and demographic characteristics” [7]. Therefore, the importance of “genetic biodemography” is expected to continue to grow in the coming years. This calls for development of appropriate methodology to get the full advantage of such rich data with diverse information. Consider, for example, the research projects related to the evaluation of the genetic effect on some time-to-event outcome, for example, risks of death or onset of diseases. The traditional approach to estimating the effect of genetic markers in such cases is to use only information on genotyped individuals ignoring the nongenotyped part of the data and the demographic structure of the sample under study. However, when genetic data are included in longitudinal studies of aging, there are two additional sources of information that can enhance genetic analyses.

First, usually genetic data are collected in longitudinal studies from participants at different ages at baseline (or at genotyping). Therefore, this provides information on the age structure of the population at the time of genotyping (or, generally, biospecimen collection). Along with follow-up data, such population age structure contains information about the effect of genetic variants on the outcome of interest (i.e., lifespan or age at onset). Indeed, in order to be genotyped, an individual has to survive until the age at biospecimen collection. Hence, if the proportion of carriers of some genetic variant increases with age (i.e., age at biospecimen collection) then this variant should favor longevity. This allows associating this genetic variant with lifespan even without the follow-up data using the “gene frequency” method [31, 32]. Therefore, using both follow-up data and data on the population age structure should provide more accurate estimates of parameters compared to the use of follow-up data of genotyped individuals alone.

Second, an additional source of information in longitudinal studies of aging that is relevant for genetic analyses is of a historical origin. It is a common situation that many long-established longitudinal studies started long before the genetic data collection became a common practice. In such studies genetic data are collected only for a subsample of participants (i.e., those who survived until the time of biospecimen collection). Budgetary restrictions can be another possibility for collecting genetic data only for a subsample of participants. In any case, information on the outcome of interest (e.g., mortality or onset of diseases) can usually be available for all individuals from the longitudinal study, both with and without genetic data. This information provides an additional reserve for increasing power and improving the accuracy of the estimates. Indeed, the group of nongenotyped individuals is a mixture of carriers/noncarriers of the same alleles or genotypes collected in the genetic data and a similar functional form of mortality rate can be assumed for the entire sample. Therefore, this information can be appropriately combined in the likelihood function with information for individuals with genetic data [33]. For example, joint analysis of genotyped and nongenotyped subsamples, along with information on ages at genotyping, revealed that female carriers of the APOE e4 allele have significantly worse survival than noncarriers of that allele in the Framingham original cohort data, whereas analysis of the genotyped subsample alone did not show significant effects [33].

Our recent simulation studies [34] illustrate that the method that combines only two sources of information, (1) ages at genotyping and (2) follow-up data for genotyped individuals, provides more accurate estimates of parameters and increases the power compared to analyses of follow-up data for genotyped individuals alone. The effect was especially noticeable in the scenario with a short follow-up. This indicates that in the studies with a long follow-up period information from follow-up makes a more valuable contribution than information hidden in the distribution of ages at genotyping. On the other hand, in studies with a short follow-up period, the distribution of ages at genotyping plays a more prominent role in differentiating allele- or genotype-specific survival patterns.

These two examples illustrate the value of implementing demographic data in genetic analyses of time-to-event outcomes which is especially important in genetic studies of longevity with generally limited sample sizes and expected moderate effect sizes.

#### 2.2.2. Biodemographic Analyses of Genetic Regulation of Lifespan

The search for genetic determinants of complex traits such as lifespan has long been in a focus of scientific research. Recent advances such as genome wide association studies (GWAS) helped in revealing hundreds of genetic variants associated with such complex traits [35]. Nevertheless, the results for longevity-related traits generally did not meet expectations because almost none of the found variants reached the genome-wide significance level [36–40]. Also, the proportions of phenotypic variance of complex traits explained by the genetic variants found in GWAS were much smaller than expected values estimated from the narrow sense heritability of these traits evaluated in the pregenomic era raising the discussion on the “missing heritability” [35, 41–44]. One limitation of the traditional GWAS is that they use the “one genetic variant at a time” paradigm ignoring the fact that many genetic factors can contribute to such complex traits and they work in concert to regulate the trait. While individual genetic factors can have minor effect on the trait, their combined influence could be substantial and significant. In [45] we showed that such influence does take place using the polygenic scores (or “genetic doses”) constructed from such genetic variants (single-nucleotide polymorphisms, SNPs). The polygenic score counts the number of “longevity alleles” contained in person’s genome that resembles the construction of frailty (or cumulative deficits) index [46–48] counting accumulation of deficits in an individual during the life course. Such scores also allow for evaluation of the additive genetic component of the lifespan [49]. The notion of the additive genetic component of a trait is well established since the pregenomic era, and it was estimated indirectly using data on related individuals. The availability of GWAS data and implementation of the polygenic scores begin the era of direct estimation for this component.

Bio demographic methods allow for investigating and interpreting age patterns of mortality rates and survival functions for carriers of different numbers of “longevity alleles” in their genomes. In [49] we used two models, the Strehler-Mildvan (SM) mortality model [50] and the “life-saving” mortality model [51], to get insight into survival and mortality patterns in subgroups of individuals carrying different numbers of “longevity alleles” (resp., having different values of polygenic scores) constructed in [45] and into possible functional roles that respective genes can play in aging, health, and lifespan. We showed that survival functions in these subgroups differ substantially and that these differences are similar to those observed in the population-level survival during the last century (known in demography as “rectangularization” of survival curves). The biodemographic models permitted interpretation of such findings that the “longevity alleles” contribute to providing the organisms with additional resilience, redundancy, and robustness, which increases their ability to withstand stresses. This confirms observations from experimental studies that regulation of lifespan involves genes responsible for stress resistance. In [52] we investigated possible biological mechanisms of the polygenic influence on lifespan. We found that the respective genes are largely involved in aging, cancer, and brain disorders. This indicates that the identified “longevity alleles” have functional relevance to aging and aging-associated diseases supporting the causal relationship between respective genes and lifespan.

The applications of the SM model described in [49] indicated that genetic factors may modify values and dynamic properties of variables describing aging-related transformations in the human body and that these modifications influence lifespan. The availability of measurements of various physiological variables collected in longitudinal studies of aging, health, and longevity allows addressing questions on how genetic variants could be related to different age dynamics of physiological variables over the life course. We will review our recent works on these topics in Section 2.3.1. Recent methodological advances in biodemography make possible evaluation of hidden components of the aging-related processes and the genetic influence on these components from age trajectories of physiological variables and mortality data collected for participants of longitudinal studies. We will discuss our recent findings related to this research area in Section 2.3.2.

#### 2.2.3. Genetic Determinants of Risks of Aging-Related Diseases: Impact of Aging-Related Processes in Changing Environment

Empirical evidence on inverse dependence between risks of diseases (see Section 2.1.3) implies that some risk factors can have the opposite effect on these diseases. Genetic trade-offs, that is, when the same allele confers the risk of one disease but protects against the other, can play a role in such dependencies. Our recent studies demonstrated the presence of such trade-offs in the gene action and also the differential effect of the same genotypes at different ages that both contribute to the observed complex dynamics of aging-related traits (such as diseases risks) in the elderly populations.

In [22] we investigated the association of the APOE e2/3/4 polymorphism with lifespan and ages at onset of cardiovascular diseases (CVD) and cancer, using data on participants of the Framingham Heart Study Offspring (FHSO) cohort. Empirical (Kaplan-Meier) estimates showed that the e4 allele carriers live shorter lives than the non-e4 allele carriers and the adverse effect was attributed to the poor survival of the e4 homozygotes, whereas the effect of the common e3/4 genotype was insignificant. However, the striking finding of this study was that the e3/4 genotype showed a pleiotropic effect with a trade-off; that is, it can predispose to earlier onset of CVD but postpone cancers to older ages, compared to the non-e4 genotypes. This genetic trade-off explains the lack of a significant effect of the e3/4 genotype on survival and this observation illustrates that genetic trade-offs appear to be an important source of confounding in studies of genetic effects on aging-related traits.

In [53] we extended these analyses for the Framingham Heart Study (FHS) original cohort to elucidate the potential role of age and gender in trade-off of the APOE e4 allele on risks of premature onset of CVD and cancer using data on two generations followed up for about 60 years. We found that in the offspring cohort the e4 allele confers risk of CVD primarily in women and can protect against cancer primarily in men of the same age. In the parental generation, the genetic trade-off is seen in different age groups with protective role of the e4 allele against cancer in older men and its detrimental role in CVD in younger women. Our study thus demonstrates that the aging-related processes in different generations (which can serve as proxies for environmental changes) can substantially impact the strength and significance of genetic effects on traits in late life. In [54], we found (using a different study, the Long Life Family Study) that biogenetic mechanisms underlying relationships among different phenotypes can function differently in successive generations or in different age groups of biologically related individuals.

In [55] we further elucidated the impact of aging-related processes in a changing environment on the role of lipid-related genes discovered in candidate gene (the APOE e2/3/4 polymorphism) and genome-wide (the APOB rsl042034 (C/T)) studies, in regulation of total cholesterol (TC) and onset of CVD in the FHS and FHSO cohorts. One important observation was that the APOE e4 allele and the APOB CC genotype can play detrimental, neutral, and protective sex-specific roles in the etiology of CVD at different ages and in different environments. Another important observation was that aging-related processes can modulate the strength of genetic associations with TC in the same individuals at different chronological ages. This study thus demonstrates the crucial role of aging-related processes in a changing environment in the genetics of health span. Ignoring such effects can produce misleading conclusions on the effects of genes on the traits of interest. For example, disregarding the role of aging erroneously nullified the significant effects of the e4 allele in this study.

All these results suggest that the role of aging-related processes in changing environment may be conceptually underestimated in current genetic association studies. Thus, as we indicated in [53], “… if we believe that a trait in late life can have genetic origin, we have to also keep in mind that (i) the same genes causing that trait can change their role with individuals’ aging; (ii) different genes can work at different chronological ages; and/or (iii) aging-related genetic effects can be altered by the environment, even in an antagonistic fashion.” One important methodological implication of these studies is that they explicitly demonstrate that standard biostatistical approaches and assumptions commonly used in genetic analyses of aging-related traits can be insufficient and new methods providing deeper insights into biological mechanisms mediating gene action are needed. More detailed analyses of existing genotype data using the new methods that utilize systemic integrative approaches could substantially advance the scientific progress in the field. Currently available longitudinal data on aging, health, and longevity contain a wealth of information on age trajectories of various physiological variables that can be used to get insight into systemic mechanisms of aging-related changes and elucidate the role of genetic component in these processes. The next Section 2.3 reviews our recent work on a special type of biostatistical models, the stochastic process model of aging, which can be used as a methodological platform for performing such systemic integrative analyses.

### 2.3. Recent Advances in Biodemographic Analyses of Longitudinal and Time-to-Event Data

#### 2.3.1. Age Trajectories of Biomarkers in Relation to Mortality and Morbidity Risks: Empirical Evidence from Longitudinal Data on Aging, Health, and Longevity

Individual age trajectories of biomarkers reflect the results of influence of different factors acting during the individual’s life course. This includes the adaptive and compensatory response in physiological systems in response to deterioration produced by the senescence process or by external disturbances. Therefore, the values and the dynamics of such biomarkers can be predictive factors for longevity and healthy lifespan. In this section, we will discuss our recent findings from the FHS data on patterns and regularities of age trajectories of biomarkers, their relation to mortality and morbidity risks. The next section will address methodological developments related to the stochastic process model that jointly analyzes the longitudinal trajectories of biomarkers and data on mortality or morbidity, taking into account several major concepts on the aging-related processes accumulated in the literature.

Although individual age trajectories of different biomarkers show substantial variability across individuals (see, e.g., [56]), their average patterns reveal remarkable regularities (see [57–59]). Some biomarkers (such as blood glucose and pulse pressure) have almost monotonic changes with increasing age. But for the majority of biomarkers (such as body mass index, total cholesterol, diastolic blood pressure, hematocrit, and pulse rate) the average trajectories are clearly nonmonotonic: after a period of increase the values of biomarkers reach their maximum value (at some sex- and biomarker-specific ages) and then decline. Importantly, these average age trajectories do not represent average biological changes developing in an aging human organism. This is because of compositional changes due to mortality selection that affect the averaging procedure and modify the population average. Individuals for whom the values of biomarker substantially deviate from their “optimal” values experience higher-than-average mortality risks and such individuals tend to die out first and drop out of the averaging procedure. In [56, 60] we illustrated this effect showing that the average trajectories of different biomarkers in individuals dying at earlier ages markedly deviate from those in long-lived individuals. Specifically, trajectories for many biomarkers in individuals dying at earlier ages increase to higher levels and/or start declining earlier, sometimes at a faster rate, thus revealing that deviant dynamics of biomarkers with age might lead to higher chances of death. Similar difference was also observed for average trajectories for individuals with long and short healthy lifespan (defined as the period of life free of cardiovascular diseases, cancer, or diabetes). Note also that average age trajectories of biomarkers for long-lived individuals (say, for those surviving until age 90 years) do not include compositional changes before that age, and, therefore, such trajectories represent average biological changes developing in an aging organism. We utilized this observation in the evaluations of “physiological norms” in our applications of the biostatistical models discussed in the next section.

In [56, 60] we also evaluated how dynamic characteristics of individual age trajectories can affect mortality risk. For biomarkers with almost monotonic age trajectories, we evaluated the effect of an initial value at age 40, the rate of change at ages 40 to 60, and the variability (i.e., deviations of observed values from those approximated by a linear function at ages 40 to 60) on mortality risk at ages 60 and older. For biomarkers with nonmonotonic changes, we also calculated the effect of various other dynamic characteristics (such as the rate of increase, age at reaching the maximal value, the rate of decline after reaching the maximum, and the variability) at biomarker- and sex-specific age intervals on subsequent mortality. The analyses showed that the evaluated dynamic characteristics of trajectories of biomarkers at middle and old ages do influence mortality risk differentiating the survival chances at older ages. For example, individuals having the slowest rate of decline after reaching the maximal level of biomarkers had the best survival chances. For the majority of dynamic characteristics and biomarkers, the effects were monotonic but in some cases the U-shape of the effect was observed with the middle tertile having the best survival chances. The effect of the dynamic characteristics on the risk of onset of “unhealthy life” (i.e., cardiovascular disease, cancer, or diabetes) was in most cases similar but less pronounced than that on mortality risks (see also [61]).

Availability of genetic information in longitudinal studies on aging allows checking whether there is a difference in the age dynamics of biomarkers in carriers of different genotypes or alleles. In [62] we evaluated average age trajectories of physiological variables (total cholesterol (TC) and diastolic blood pressure (DBP)) in long-lived female and male carriers and noncarriers of the APOE e4 allele in the FHS data. We also calculated average age trajectories of these physiological variables for female and male carriers and noncarriers of the e4 allele who survived until different ages. We found that the average age trajectories of TC and DBP in individuals dying at earlier ages markedly deviate from those of the long-lived groups and these patterns differ for carriers and noncarriers of the e4 allele. Long-lived individuals, compared to shortlived ones, have consistently higher levels and a less steep decline of both TC and DBP at old ages (65+) when such levels naturally go down in aging human organism. This observation is in line with our findings in [56, 60, 61] that individuals with the slowest decline in biomarkers had the best survival chances.

Genetic effects on lifespan and survival are mediated by a number of intermediate variables whose effects are integrated in the values of physiological variables. In [52] we showed that lifespan of the participants of the Original FHS cohort was positively associated with the “dose” of 27 individually selected genetic variants (called “longevity” alleles) each having a small positive effect on lifespan. Since the genetic dose index was associated with lifespan and the values of physiological variables measured in the Original FHS cohort were also associated with lifespan, it is natural to expect that the genetic dose index would show an association with the age trajectories of these physiological variables. In [59] we illustrated that such association does exist comparing age trajectories of physiological variables in the groups of individuals carrying different number of “longevity” alleles (<14 versus >=14). We found that the 27 SNP alleles associated with lifespan influence the age trajectories of physiological indices in this study in complex ways. This includes (1) the parallel shift of the age trajectory of an index to the right (for those carrying larger numbers of “longevity” alleles); (2) the same age at reaching the maximal value but different maximal levels; and (3) different rates of decline after reaching the maximum. Such observations indicate that some of the 27 “longevity” alleles may modulate predisposition to particular diseases, while some may regulate the rate and onset of physiological aging changes, and some other alleles may have pleiotropic influence on respective traits. These findings confirmed our earlier analysis of the functional effects of genes linked to these 27 SNPs, where we found that such genes are deeply involved in physiological aging and common diseases [52].

The analyses described above confirmed that genetic influence on lifespan is realized through dynamic mechanisms regulating changes in physiological variables during the life course. However, various hidden aging-related processes may jointly contribute to such effects and they cannot be evaluated from available data using conventional approaches. Examples of such processes include decline in adaptive capacity of an organism, decline in resistance to stresses, and age-related changes in “dynamic set-point trajectory” which the trajectories of biomarkers are forced to follow by the processes of allostatic adaptation. In the next section we present our recent methodological developments and applications of the advanced biostatistical models that allow for evaluating such “hidden components” of aging-related changes indirectly from age trajectories of physiological variables and evaluate genetic component in aging-related processes.

#### 2.3.2. Patterns of Aging-Related Changes in Relation to Mortality and Morbidity Risks: Insights from Statistical Modeling

Longitudinal data on aging, health, and longevity that contain individual measurements of biomarkers at different ages along with follow-up data on health and survival status provide a valuable source of information for evaluation of the impact of dynamics of biomarkers on mortality and morbidity risks. Traditional approaches such as the Cox proportional hazards model with time-dependent covariates have certain limitations in applications to biomarkers that have measurement errors and biological variation [63, 64]. Joint models [65–70] developed in the recent biostatistical literature are useful in many applications. However, they are of a limited use in applications aiming at research on aging because such models typically lack specific parameters that can be biologically interpreted it the context of aging. Analyses of longitudinal data on aging require special methodological approaches that reflect knowledge and evidence accumulated in the literature on aging.

An important class of models for joint analyses of longitudinal and time-to-event data that became known as the stochastic process models (SPM) or, alternatively, the quadratic hazard models, is an example of such approach. They are based on biologically justified assumption of a quadratic hazard (i.e., U- or J-shaped) as a function of biomarkers at specific age. Their advantage is that they incorporate important theoretical concepts capturing fundamental features of aging-related changes in an organism that are available in the literature. This includes the notion of allostatic load [71], the decline in adaptive capacity (homeostenosis) [72–75], the decline in resistance to stresses [50, 76–78], aging-related physiological norms, and heterogeneity in longitudinal data. The original version of SPM was suggested in [79] and since then it was further developed and applied in many contexts. Here we review its recent developments and applications to investigate the relationships between the longitudinal dynamics of biomarkers and mortality and morbidity risks.

In [56, 58, 60] we applied the SPM [79] to data on age trajectories of several biomarkers (such as blood glucose, body mass index, diastolic blood pressure, hematocrit, pulse pressure, pulse rate, and serum cholesterol) collected for participants of the Framingham original cohort and data on mortality and onset of “unhealthy life” (defined as onset of cardiovascular diseases, cancer, or diabetes). We observed several regularities in the estimates of the models applied to different biomarkers as well as systematic differences in the patterns of models’ components for females and males. Specifically, we found that the baseline hazard for females and males follows the same pattern for all considered biomarkers; however, it is always lower but increases faster with age in females compared to males. For the majority of biomarkers, the U-shape of the hazard rates (both mortality and onset of “unhealthy life”) considered as a function of biomarker at specific ages narrows with age. Also the width of the U-shape is narrower in males than in females, and the narrowing of the U-shape with age is faster in males than in females. This means that at old ages males generally pay a higher “price” for deviations from the optimal values of biomarkers compared to females suggesting that males have generally lower resistance to stresses than females, and the rate of decline in stress resistance is faster in males than in females. We also revealed the decline in adaptive capacity with age for many biomarkers; that is, more time is needed for the trajectories of biomarkers to return to the “dynamic set-point trajectory” in case of disturbances at older ages than at younger ages. We also showed that this “dynamic set-point trajectory” which the trajectories of biomarkers are forced to follow is different from the trajectories minimizing risks of death or onset of “unhealthy life” (and which can be interpreted as age-dependent physiological norms). Such persistent deviations from the “norm” characterize the effects of allostatic adaptation and the magnitudes of such deviations for each physiological variable can be associated with the components of allostatic load leading to increased chances of development of the diseases or death. All these components combined affect the patterns of mortality and incidence rates and contribute to the differences between the rates in females and males. Importantly, these aging-related characteristics (such as stress resistance, adaptive capacity, and physiological norms) are hidden in the individual longitudinal trajectories of physiological variables and can be analyzed only indirectly using statistical modeling. The SPM is thus a useful approach for evaluating hidden components of aging-related changes from longitudinal data that have not been analyzed before using conventional approaches. One important future development in the SPM methodology is to implement the recently developed measure of physiological dysregulation based on the statistical distance of biomarker profiles [80]. This model would have computational advantages as it allows for working with multiple biomarkers in the one-dimensional framework It also provides a useful approach to evaluating how such a measure of physiological dysregulation is related to different aging-related characteristics in the traditional SPM.

Availability of genetic data for participants of a longitudinal study makes it possible to evaluate genetic component in the respective aging-related processes. One possibility is to apply the original SPM [79] to subsamples of carriers and noncarriers of some allele or genotype. However, nongeno-typed participants who still have data on other relevant outcomes (i.e., longitudinal measurements of biomarkers and follow-up data on mortality and morbidity) provide an additional reserve to increase the accuracy and power in analyses of genetic effects on longitudinal and time-to-event outcomes. This can be done using the modified SPM that was named the “genetic stochastic process model,” or the “genetic SPM” [81]. In [62] we evaluated effects of the APOE polymorphism and age trajectories of physiological variables (total cholesterol and diastolic blood pressure) on mortality risk in the Framingham original cohort. We observed different patterns of aging-related characteristics (adaptive capacity, decline in stress resistance, mean allostatic trajectories, and the baseline hazard rate) in carriers and noncarriers of the APOE e4 allele. These differential patterns may contribute to the observed differences between the shapes of survival functions and average age trajectories of respective physiological variables in carriers and noncarriers of the e4 allele as well as differences in these characteristics between females and males. This calls for additional studies to understand the underlying determinants of such differences in aging-related characteristics. Taking into account the observed trade-offs in the effects of the APOE polymorphism on the ages at onset of aging-related diseases [22], one important development is to apply the method to data on incidence of such diseases and cause-specific mortality. This can reveal possible tradeoffs in the effects of the APOE polymorphism on regularities of different aging-related characteristics that can be masked in the analyses of the total mortality data.

The approach can be also applied to analyze the regularity of aging-related changes in carriers of different SNP alleles evaluated from GWAS data on respective outcomes such as longevity. In [59] we applied the SPM to data on the two groups of genotyped individuals from the Framingham original cohort, those carrying <14 and >=14 alleles out of the 27 “longevity” alleles selected in [52]. We showed that these two groups differ substantially in terms of the baseline hazard rates, the adaptive capacity, and the average allostatic trajectory. Specifically, the baseline hazard (i.e., the hazard summarizing the effect of all factors except respective physiological variable) is lower in carriers of a larger number of “longevity” alleles (>=14) compared to carriers of a smaller number of such alleles (<14). Also the differences in survival chances in the two groups can be explained by differences in other characteristics such as adaptive capacities and average allostatic trajectories: carriers of a larger number of “longevity alleles” have significantly better adaptive capacity; that is, in the group carrying a larger number of longevity alleles, the trajectories of respective physiological variables return faster to the average “allostatic” trajectories that organisms are forced to follow than they do in the individuals carrying a smaller number of such “longevity” alleles. These observations allowed us to conclude that the average aging-related changes in the respective physiological variables (see the narrative in the previous section) can be driven by hidden components of aging changes and that genetic factors play an important role in this process.

The ability of the SPM to estimate hidden components of aging changes in humans as presented above has already proved that this approach is a useful tool for performing comprehensive analyses of available longitudinal data that allows incorporating relevant biological knowledge about aging into statistical analyses. Nevertheless, the approach still can be further extended in many important directions. In [82, 83] we reviewed recent developments in the SPM methodology. We also further extended the approach to include the dependence of all components of the model on observed covariates. This is an important step that makes the model more “personalized.” Such modification allows for addressing important questions on dependence of the hidden components of aging on different (time-independent) variables, for example, sociodemographic variables or genetic “doses” such as those described above. Further directions towards personalization involve developing a more general methodology, which could incorporate individualized components such as “norms” or adaptive responses. In [84] we presented a generalized version of SPM with competing risks. This version assumes conditional independence of cause-specific times to death, given a stochastic process with two mutually dependent continuous and jumping components. This assumption about conditional independence is much weaker than the traditional assumption on independent competing risks conventionally used in analyses. This is a significant step forward in the methodology because generally cause-specific mortality risks are mutually dependent and thus it is important to avoid the marginal independence assumption when appropriate data (and methods) are available.

A comprehensive approach for joint analyses of data on individual health histories, age trajectories of physiological or biological variables, and mortality has been presented in [85]. When individual health trajectory needs to be taken into account, the coefficients of the stochastic differential equations describing physiological aging in SPM will depend on the jumping process describing changes in health status. The comprehensive model of human aging, health, and mortality suggested in [85] has both jumping and continuous components that jointly have the Markov property. The jumping component represents fast changes in health status, and the continuous component describes slower individual physiological aging. The important practical value of the developed approach is that it provides the possibility to jointly analyze data with different structures (e.g., discrete-time observations of continuously changing physiological variables with unobserved changes in health status, or unmeasured physiological variables but observed health transitions, or a combination of the above) within the same methodological framework [86, 87]. Such joint analysis of combined data has an obvious advantage because separate analyses of each such “incomplete” dataset cannot provide estimates of all model’s parameters. Thus this combined model is a unique tool to connect trajectories of physiological variables with health status and mortality that has parameters interpretable in terms of properties of respective aging-related processes. Further developments with this methodology should deal with individualized dynamic mechanisms involved in regulation of aging-related changes in each study participant that will contribute to development of personalized preventive and treatment strategies.

## 3. Conclusions

In this paper, we summarized our recent publications related to different methodological and applied aspects in biodemography This covers three major topics: reconstruction of age patterns of incidence rates and other characteristics using Medicare claims data, genetic and genomic biodemography in the search on genetic determinants of longevity and health, and development of biodemographic models for joint analyses of time-to-event data and longitudinal measurements of biomarkers collected in longitudinal studies on aging. Here we indicate some limitations of the current body of work conducted and highlight a few possible future directions of research.

Section 2.1 shows how Medicare claims data can be used to evaluate population-based patterns of incidence of geriatric diseases and other related characteristics as well as their time trends. These studies have substantial practical importance *per se*, but they also have a potential for further directions in the area of predictive modeling. The body of work conducted so far and summarized in the publications reviewed in Section 2.1 serves as a background and “building blocks” that can be used in a comprehensive predictive modeling of mortality and health in the elderly population that takes into account the dynamics and patterns of all these characteristics in their mutual connection.

Finding genetic determinants of health and longevity are an immense topic and our studies reviewed in Section 2.2 highlight just a few aspects of this research agenda from biodemographic perspectives. One important practical aspect is that application of the biodemographic approach that combines follow-up data and demographic structure of the population at the time of genotyping increases the power compared to analyses of follow-up data for genotyped individuals alone. However, at present, such an approach has limitations because it assumes that initial allele/genotype frequencies do not depend on any covariates and that all individuals in a sample are independent. Therefore, important future directions of this approach are to allow for dependence of the initial frequencies on observed covariates (such as birth cohorts, other sociodemographic indicators or principal components traditionally used in genetic analyses) and generalize the approach to working with related individuals.

Current applications of polygenic scores reviewed in Section 2.2.2 have a limited focus on “longevity alleles,” that is, those favoring longevity. However, survival advantage may come not only from the presence of “longevity alleles” in the genome but also from the absence of “frailty alleles,” that is, those having detrimental effect on longevity. Analyses of polygenic scores counting the number of “frailty alleles” as well as the joint analyses of “longevity” and “frailty” scores and their relation to risks of aging-related diseases and longitudinal dynamics of biomarkers are possible future directions of these analyses.

Our recent studies revealing genetic trade-offs, pleio-tropic effects, and differential effects of genes at different ages (reviewed in Section 2.2.3) were limited to specific candidate genes such as APOE. Nevertheless, the natural further step is to extend such analyses to perform “life course” GWAS to identify pleiotropic and specific genetic underpinnings of such traits as lifespan, risks of major diseases, and longitudinal dynamics of physiological state of human body. However, this challenging task requires not only extensive data but also appropriate methodology that would permit integrative analyses of factors and mechanisms involved in regulation of all these mutually related processes during individuals’ life course using a wealth of available information. The stochastic process models reviewed in Section 2.3 which incorporate state-of-the-art concepts of systems biology in aging research provide a suitable methodological framework to perform such integrative analyses.

Another yet largely unexplored area of applications of the stochastic process models is their use in forecasting of mortality and health in the elderly US population. The most comprehensive form of the model can incorporate information about genetic and nongenetic factors including pleiotropic, polygenic, and age-specific effects of genes on health and survival, as well as dynamic mechanisms of aging-related changes evaluated from longitudinally measured physiological variables. The approach also allows for combining data from different sources with different structures which is especially valuable when no single dataset contains all required information to fit such a comprehensive model.
